# Data sources and applied methods for paclitaxel safety signal discernment

**DOI:** 10.3389/fcvm.2023.1331142

**Published:** 2024-02-23

**Authors:** Laura Elisabeth Gressler, Erika Avila-Tang, Jialin Mao, Alejandra Avalos-Pacheco, Fadia T. Shaya, Yelizaveta Torosyan, Alexander Liebeskind, Madris Kinard, Christina D. Mack, Sharon-Lise Normand, Mary E. Ritchey, Danica Marinac-Dabic

**Affiliations:** ^1^ Office for Clinical Evidence and Analysis, United States Food and Drug Administration, Silver Spring, MD, United States; ^2^ Division of Pharmaceutical Evaluation and Policy, University of Arkansas for Medical Sciences, Little Rock, AR, United States; ^3^ Department of Population Health Sciences, Weill Cornell Medicine, New York, NY, United States; ^4^ Applied Statistics Research Unit, Faculty of Mathematics and Geoinformation, TU Wien, Vienna, Austria; ^5^ Harvard-MIT Center for Regulatory Science, Harvard Medical School, Boston, MA, United States; ^6^ School of Pharmacy, University of Maryland Baltimore, Baltimore, MD, United States; ^7^ Division of Clinical Evidence and Analysis 3, United States Food and Drug Administration, Silver Spring, MD, United States; ^8^ Office of Product Evaluation and Quality, United States Food and Drug Administration, Silver Spring, MD, United States; ^9^ Device Events, York, PA, United States; ^10^ IQVIA Real World Solutions, Research Triangle Park, Raleigh, NC, United States; ^11^ Department of Epidemiology, Gillings School of Global Public Health, University of North Carolina, Chapel Hill, NC, United States; ^12^ Department of Health Care Policy, Harvard Medical School, Boston, MA, United States; ^13^ Department of Biostatistics, Harvard School of Public Health, Boston, MA, United States; ^14^ Med Tech Epi, Philadelphia, PA, United States; ^15^ Center for Pharmacoepidemiology and Treatment Science, Rutgers University, New Brunswick, NJ, United States

**Keywords:** paclitaxel-coated devices, stents, balloons, data quality, signal discernment, latemortality

## Abstract

**Background:**

Following the identification of a late mortality signal, the Food and Drug Administration (FDA) convened an advisory panel that concluded that additional clinical study data are needed to comprehensively evaluate the late mortality signal observed with the use of drug-coated balloons (DCB) and drug-eluting stent (DES). The objective of this review is to (1) identify and summarize the existing clinical and cohort studies assessing paclitaxel-coated DCBs and DESs, (2) describe and determine the quality of the available data sources for the evaluation of these devices, and (3) present methodologies that can be leveraged for proper signal discernment within available data sources.

**Methods:**

Studies and data sources were identified through comprehensive searches. original research studies, clinical trials, comparative studies, multicenter studies, and observational cohort studies written in the English language and published from January 2007 to November 2021, with a follow-up longer than 36 months, were included in the review. Data quality of available data sources identified was assessed in three groupings. Moreover, accepted data-driven methodologies that may help circumvent the limitations of the extracted studies and data sources were extracted and described.

**Results:**

There were 39 studies and data sources identified. This included 19 randomized clinical trials, nine single-arm studies, eight registries, three administrative claims, and electronic health records. Methodologies focusing on the use of existing premarket clinical data, the incorporation of all contributed patient time, the use of aggregated data, approaches for individual-level data, machine learning and artificial intelligence approaches, Bayesian approaches, and the combination of various datasets were summarized.

**Conclusion:**

Despite the multitude of available studies over the course of eleven years following the first clinical trial, the FDA-convened advisory panel found them insufficient for comprehensively assessing the late-mortality signal. High-quality data sources with the capabilities of employing advanced statistical methodologies are needed to detect potential safety signals in a timely manner and allow regulatory bodies to act quickly when a safety signal is detected.

## 1 Introduction

Drug-coated balloons (DCBs) and drug-eluting stents (DESs) are frequently used in revascularization procedures among patients diagnosed with atherosclerosis. More specifically, devices coated or eluting paclitaxel have been associated with a decreased risk of restenosis and reintervention (1, 2). Paclitaxel hinders scar tissue from forming in the treated vessel, thus preventing restenosis. On December 18, 2018, Katsanos et al., published a meta-analysis of long-term mortality rates in 28 randomized controlled trials (RCTs) in subjects treated with paclitaxel-coated devices, compared to uncoated control devices, in the femoral or popliteal arteries (3). The meta-analysis included publicly available data from clinical trials that evaluated DCB and DES. The clinical trials included devices available within and outside the United States (US) and captured 1-, 2-, and 5-year study-level mortality data. The authors concluded that the risk of death was significantly greater in patients treated with DCB and DES devices than the control devices at each assessed timepoint.

In June 2019, the Food and Drug Administration (FDA) convened a public advisory committee meeting to discuss late mortality signal and provide recommendations on the necessary regulatory actions (4). The committee reviewed the existing evidence on the use of DCB and DES and noted that the studies thus far, including the meta-analysis, suffer from critical limitations. These limitations include the lack of patient-level data, cause of death information, detailed paclitaxel dose information, and information regarding missing data and follow-up data. Given these limitations, the panel and FDA agreed that additional clinical study data are needed to comprehensively evaluate the late mortality signal.

High-quality data with applied appropriate statistical methods are needed to accurately ascertain a signal from a device that may not be performing as anticipated in premarket clinical trials. While RCTs and other clinical studies provide foundational evidence on the safety and effectiveness of a device, real-world data sources that capture the clinical use of these devices among the broader population can provide further insight into the devices' performance. Even when available, high-quality data sources are not sufficient for the assessment of devices. Appropriate statistical methods relevant to the leveraged data sources need to be employed to minimize bias and produce the needed evidence to inform regulatory and clinical decision making.

The objective of this review is thus to (1) identify and summarize the existing clinical and cohort studies assessing paclitaxel-coated DCBs and DESs, (2) describe and determine the quality of the available data sources for the evaluation of these devices, and (3) present methodologies that can be leveraged for proper signal discernment within available data sources.

## 2 Methods

### 2.1 Identification of studies

Comprehensive searches conducted in MEDLINE, EMBASE, and clinicaltrials.gov identified relevant completed or ongoing studies and data sources. The search strategy used the following terms: “paclitaxel-coated balloon,” “paclitaxel-eluting stent,” “paclitaxel drug-coated balloon,” “paclitaxel drug-eluting stent,” “DCB,” and “DES.” Studies initiated, and data sources available from January 2007 to November 2021 were identified. Including studies that have been initiated but not yet completed allows for the comprehensive assessment of existing collected data and upcoming soon-to-be available data. Bibliographies were cross-referenced for additional citations that did not arise in the original search. Original research, clinical trials, comparative studies, multicenter studies, and observational cohort studies written in the English language and evaluating the paclitaxel-coated balloons or paclitaxel-eluting stents were included in the review. Identified studies or data sources with a follow-up duration of fewer than 36 months were excluded.

### 2.2 Data sources quality assessment

Data quality considerations from regulatory, international societies, and initiative guidance were reviewed. These documents indicate the need for data relevance, reliability, and robustness to have sufficient quality to be “fit for purpose” and address research questions.

Based on the recommended quality assessment criteria for real-world evidence (RWE) and considerations for signal discernment regarding a long-term safety outcome, we determined that data quality could be assessed in three groupings: (1) availability of critical data elements, (2) study design for the original data collection or data source analysis, and (3) four questions specific to data quality assessment (5–9). Critical data elements included data related to device exposure, mortality, lifestyle, comorbidities, medications, procedures, and physical status/frailty. Additionally, the number of patients in the study at the time of the procedure and the 3 and 5 years following the procedure were recorded. Questions related to the study design, data quality, to the objectives of the original study, generalizability of findings, and the underlying population from the original study were included in the assessment.

The four additional questions specific to data quality were:

• Were there any changes in variable capture or by study site over the study period? This question clarifies whether there were substantial changes in an RCT protocol or transitions in coding elements for RWE (e.g., transition from ICD-9-CM to ICD-10-CM) during the study period.
• What was the timing between points of data capture? This question clarifies whether there were extended time periods between data collection points.
• Will the data source owners (or researchers conducting signal refinement) be able to utilize patient-level data for additional analysis? This question clarifies whether investigators could perform additional analyses on the data collected.
• Do the data source owners (or researchers conducting signal refinement) have the ability to obtain and utilize clinical records for patients included in the data source? This question clarifies whether the data is accessible for validation purposes and further hypothesis testing with covariates not collected in the original study.

The authors assessed these aspects of data quality for each RCT, single-arm, and RWE data source.

### 2.3 Data-driven methodologies for the assessment of identified data sources

Following the extraction of relevant studies and data sources, data-driven methodologies commonly used within the various data types and established within the statistical, regulatory, and clinical communities that may help circumvent the limitations of the respective studies and data sources were identified.

## 3 Results

There were 39 studies and data sources identified. This included 19 RCTs, nine single-arm studies, eight registries, three administrative claims, and electronic health records. All the included studies and data sources are summarized in Table 1.

**Table 1:** Data sources for paclitaxel signal discernment.

Study/ Data Source Identifier ^a^ Completion Date	Data Type/ Study Design Geography	Paclitaxel Device Control Treatment	Sample Size	Length of Follow-up	Objectives (primary endpoint(s)) Generalizability to US population	Key RAPID Core Data Elements Collected^b^
Randomized Clinical Trials (Premarket)						
Thunder NCT00156624 2007	RCT (Premarket) Germany	*PTX-coated balloon:* Cotavance Balloon (Bavaria Medizin) *Control treatment:* POBA	Total: 102 Exposed:48 Controls: 54	60 months (planned after 6-month results showed differences between groups) 3 years: No assessment 5 years: PTX: 44 (78%) Controls: 29 (54%) Differential LTFU: (1 site closure (2 PTX and 7 controls)	Efficacy (late lumen loss) Limited generalizability	– Mortality outcome: all-cause – Lifestyle: SMK – Comorbidities: DM, IDDM, HTN – Disease characteristics: Rutherford – Lesion characteristics: LL, CTO, RVD, HxIL – Medications: aspirin, P2Y12 receptor blockers
Zilver PTX NCT00120406 2014	RCT (Premarket) US Germany, Japan	*PTX-coated stent:* Zilver PTX (Cook Medical) *Control treatment:* POBA	Total: 479 Exposed: 241 Controls: 238 (120 with acute PTA failure randomized to DES (*n*=61) or BMS (*n*=59))	Follow-up: 60 months Combined follow-up: ≈6.4% per year	Safety and Efficacy (event-free survival^d^, primary patency) Limited generalizability	– Mortality outcome: all-cause – Lifestyle: BMI, SMK – Comorbidities: DM, IDDM, HTN, CAD, CHF, RD/D, pulmonary disease – Disease characteristics: Rutherford – Lesion characteristics: LL, CTO, RVD, HxIL, TASC – Medications: aspirin, P2Y12 receptor blockers
REAL PTX NCT01728441 2017	RCT (Premarket) Belgium, Germany	*PTX-coated stent:* Zilver PTX (Cook Medical) *PTX-coated balloons:* IN.PACT Admiral (Medtronic)	Exposed: 150	Follow-up: 36 months 3 years: PTX DES: 51 (68%) PTX DCB: 54 (72%)	Safety and Efficacy (peak systolic velocity ratio, TLR) Limited generalizability	– Mortality outcome: all-cause, CV-related – Lifestyle: BMI, SMK – Comorbidities: DM, IDDM, HTN, CAD, CHF, RD/D – Disease characteristics: Rutherford – Lesion characteristics: LL, CTO, RVD – Medications: aspirin, P2Y12 receptor blockers
IN.PACT SFA I and SFA II NCT01175850 NCT01566461 2018	RCT (Premarket) US Germany	*PTX-coated balloon:* IN.PACT Admiral (Medtronic) *Control treatment:* POBA	Total: 331 Exposed: 220 Controls: 111	Follow-up: 60 months 3 years: PTX: 195 (89%) Controls: 101 (91%) 5 years: PTX: 184 (84%) Controls: 98 (88%)	Safety and Efficacy (primary patency, safety composite^e^) Limited generalizability	– Mortality outcome: all-cause – Lifestyle: BMI, SMK – Comorbidities: DM, IDDM, HTN, CHD, RD/D – Disease characteristics: Rutherford, prior amputation – Lesion characteristics: LL, CTO, RVD, HxIL, TASC – Medications: ATT, aspirin, P2Y12 receptor blockers
IN.PACT SFA Japan NCT01947478 2018	RCT (Premarket) Japan	*PTX-coated balloon:* IN.PACT Admiral (Medtronic) *Control treatment:* POBA	Total: 100 Exposed: 68 Controls: 32	Follow-up: 36 months 3 years: PTX: 68 (68%) Controls: 32 (32%)	Safety and Efficacy (primary patency, safety composite^f^) Limited generalizability	– Mortality outcome: all-cause – Lifestyle: BMI, SMK – Comorbidities: DM, IDDM, HTN, CAD, RD/D – Disease characteristics: Rutherford, prior amputation – Lesion characteristics: LL, CTO, RVD, HxIL, TASC – Medications: ATT, aspirin, P2Y12 receptor blockers
LEVANT 2 NCT01412541 2018	RCT (Premarket) US Austria, Belgium, Germany	*PTX-coated balloon:* Lutonix (CR Bard) *Control treatment:* POBA	Total: 532 Exposed: 372 (316 + 56 roll-in subjects) Controls:160	Follow-up: 60 months 3 years: PTX: 320 (86%) Controls: 139 (87%) 5 years: PTX: 306 (82%) Controls: 133 (83%)	Safety and Efficacy (primary patency, safety composite^g^) Limited generalizability	– Mortality outcome: all-cause, CV-related – Lifestyle: BMI, SMK – Comorbidities: DM, IDDM, HTN, CAD, CHF, RD/D – Disease characteristics: Rutherford – Lesion characteristics: LL, CTO, RVD, HxIL, TASC – Medications: ATT, aspirin, P2Y12 receptor blockers, statins
RANGER SFA NCT02013193 2019	RCT (Premarket) Austria, France, Germany	*PTX-coated balloons:* RANGER (Boston Scientific) *Control treatment:* POBA	Total: 105 Exposed: 71 Controls: 34	Follow-up: 36 months 3 years: Unknown	Efficacy (late lumen loss) Limited generalizability	– Mortality outcome: all-cause – Lifestyle: SMK – Comorbidities: DM, HTN, CAD, CHF, RD/D, COPD – Disease characteristics: Rutherford, prior amputation – Lesion characteristics: LL, CTO, RVD, HxIL, TASC – Medications: aspirin, P2Y12 receptor blockers
ILLUMENATE EU RCT NCT01858363 2020	RCT (Premarket) Germany	*PTX-coated balloon:* Stellarex (Spectranetics) *Control treatment:* POBA	Total: 294 Exposed: 222 Controls: 72	Follow-up: 60 months 3 years: 3.7% 5 years: Not available	Safety and Efficacy (primary patency, safety composite^h^) Limited generalizability	– Mortality outcome: all-cause, CV-related – Lifestyle: BMI, SMK – Comorbidities: DM, HTN, CAD, CHF, RD/D, COPD – Disease characteristics: Rutherford – Lesion characteristics: LL, CTO, RVD, HxIL – Medications: ATT, aspirin, statins
ILLUMENATE RCT/PAS NCT03421561 2020	RCT (Premarket) US Austria	*PTX-coated balloon:* Stellarex (Spectranetics) *Control treatment:* POBA	Total: 400 Exposed: 300 Controls: 100	Follow-up: 60 months 3 years: 2.3% 5 years: PTX: 184 (84%) Controls: 98 (88%)	Safety and Efficacy (primary patency, safety composite^h^) Limited generalizability	– Mortality outcome: all-cause, CV-related – Lifestyle: BMI, SMK – Comorbidities: DM, HTN, CAD, CHF, RD/D, COPD – Disease characteristics: Rutherford – Lesion characteristics: LL, CTO, RVD, HxIL – Medications: ATT, aspirin, P2Y12 receptor blockers, statins
IMPERIAL NCT02574481 2022	RCT (premarket) US Austria, Belgium, Canada, Germany, Japan, New Zealand	*PTX-coated stents:* Eluvia (Boston Scientific) Zilver PTX (Cook Medical)	Exposed: 524	Follow-up: 60 months 3 years: Not available	Safety and Efficacy (primary patency, safety composite^i^) Limited generalizability	– Mortality outcome: all-cause – Lifestyle: BMI, SMK – Comorbidities: DM, IDDM, HTN, CAD, CHF, RD/D, COPD – Disease characteristics: Rutherford – Lesion characteristics: LL, CTO, RVD, HxIL (for target limb or vessel, not target lesion), TASC – Medications: ATT, aspirin, P2Y12 receptor blockers
RANGER II SFA NCT03064126 2023	RCT (Premarket) US Austria, Canada, Japan, Belgium, New Zealand	*PTX-coated balloon:* RANGER (Boston Scientific) *Control treatment:* POBA	Total: 440 Exposed: 330 Controls: 110	Intended follow-up: 60 months 3 years: Not reached yet	Safety and Efficacy (primary patency, safety composite^i^) Limited generalizability	– Mortality outcome: all-cause – Lifestyle: BMI, SMK – Comorbidities: DM, IDDM, HTN, CAD, CHF, RD/D, COPD – Disease characteristics: Rutherford – Lesion characteristics: LL, CTO, RVD, HxIL (for target limb or vessel, not target lesion), TASC – Medications: ATT, aspirin, P2Y12 receptor blockers
Compare I NCT02701543 2023	RCT (Premarket) Germany	*PTX-coated balloons:* RANGER (Boston Scientific) IN.PACT Admiral (Medtronic)	Total: 414 Exposed: 207 Controls: 207	Intended follow-up: 60 months 3 years: Not reached yet	Safety and Efficacy (primary patency, safety composite^j^) Limited generalizability	– Mortality outcome: all-cause – Lifestyle: BMI, SMK – Comorbidities: DM, HTN, CAD, RD/D, COPD – Disease characteristics: Rutherford – Lesion characteristics: LL, CTO, RVD, HxIL – Medications: aspirin, P2Y12 receptor blockers, statins (baseline)
TRANSCEND NCT03241459 2024	RCT (Premarket) US and 10 other countries	*PTX-coated balloon:* SurVeil (SurModics)^c^ *Control treatment:* POBA	Total: 446 Exposed: 223 Controls: 223	Intended follow-up: 60 months 3 years: Not reached yet	Safety and Efficacy (primary patency, safety composite^e^) Limited generalizability	– Mortality outcome: all-cause – Lifestyle: BMI, SMK – Comorbidities: DM, IDDM, HTN, CAD, CHF, RD/D – Disease characteristics: Rutherford, prior amputation – Lesion characteristics: LL, CTO, RVD, HxIL – Medications: ATT
XPEDITE NCT02936622 2024	RCT (premarket) Germany, New Zealand	*PTX-coated stents:* Zilver PTX Zilver PTX (slower-dissolving PFPC) Zilver PTX (higher-dose PFPC) (Cook Medical)	Exposed: 176	Follow-up: 60 months 3 years: Not available	Efficacy (percent diameter stenosis) Limited generalizability	– Mortality outcome: all-cause – Disease characteristics: Rutherford
SIRONA NCT04475783 2028	RCT Austria, Germany	*PTX-coated balloons* *Sirolimus-coated balloon* Magic Touch (Concept Medical)	Total: 478 Exposed: 239 Controls: 239	Follow-up: 60 months	Safety and Efficacy (primary patency, safety composite^j^ Limited generalizability	– Mortality outcome: all-cause – Disease characteristics: Rutherford
Randomized Clinical Trials (Postmarket)						
SWEDEPAD 1 NCT02051088 2021	Registry-based RCT (Postmarket) Sweden	*PTX-coated balloons* *PTX-eluting stents* *Control treatment:* POBA, BMS	Total: 2,400 Exposed: 1,200 Controls: 1,200	Follow-up: 60 months 3 years: Not available	Effectiveness (amputation)	– Mortality outcome: all-cause – Lifestyle: SMK – Comorbidities: DM, HTN, CAD, RD/D, COPD – Disease characteristics: Rutherford – Lesion characteristics: CTO, HxIL, TASC
SWEDEPAD 2 NCT02051088 2021	Registry-based RCT (Postmarket) Sweden	*PTX-coated balloons* *PTX-eluting stents* *Control treatment:* POBA, BMS	Total: 1,333 Exposed: 667 Controls: 666	Follow-up: 60 months 3 years: Not available	Effectiveness (quality of life)	– Mortality outcome: all-cause – Lifestyle: SMK – Comorbidities: DM, HTN, CAD, RD/D, COPD – Disease characteristics: Rutherford – Lesion characteristics: CTO, HxIL, TASC
ZilverPass NCT01952457 2022	RCT (postmarket) Belgium	*PTX-coated stent:* Zilver PTX (Cook Medical) *Control treatment:* Prosthetic bypass graft	Total: 220 Exposed: 113 Controls: 107	Follow-up: 60 months 3 years: Not available	Efficacy (primary patency) Limited generalizability	– Mortality outcome: all-cause – Lifestyle: BMI, SMK – Comorbidities: DM, IDDM, HTN, CAD, RD/D – Disease characteristics: Rutherford – Lesion characteristics: LL, CTO, RVD, TASC
EMINENT RCT NCT02921230 2025	RCT (Postmarket) 10 European countries	*PTX-coated stent:* Eluvia (Boston Scientific) *Control treatment:* BMS	Total: 775 Exposed: ≈517 Controls: ≈258	Intended follow-up: 60 months 3 years: Not reached	Effectiveness (primary patency) Limited generalizability	– Mortality outcome: all-cause – Lifestyle: BMI, SMK – Comorbidities: DM, IDDM, HTN, CAD, CHF, RD/D, COPD – Disease characteristics: Rutherford – Lesion characteristics: LL, CTO, RVD, HxIL (for target limb or vessel, not target lesion), TASC – Medications: ATT, aspirin, P2Y12 receptor blockers
Single-arm and cohort studies						
Zilver PTX and Flex Japan PMS NCT02254837 2018	Single-arm trial Japan	*PTX-coated stent:* Zilver PTX (Cook Medical)	Exposed: 907	Follow-up: 60 months 3 years: unknown 5 years: unknown	Safety (stent fracture and AEs) Limited generalizability	– Mortality outcome: all-cause – Lifestyle: SMK – Comorbidities: DM, IDDM, HTN, CAD, RD/D, pulmonary disease – Disease characteristics: Rutherford – Lesion characteristics: LL, CTO, RVD, HxIL, TASC – Medications: ATT, aspirin, P2Y12 receptor blockers, statins
Lutonix DCB Long Lesions NCT02013271 2018	Single-arm trial Austria, Belgium, France, Germany, Switzerland	*PTX-coated balloon:* Lutonix (CR Bard)	Exposed: 125	Follow-up: 36 months	Safety and Efficacy (primary patency, safety composite^k^) Limited generalizability	– Mortality outcome: all-cause – Lifestyle: BMI, SMK – Comorbidities: DM, IDDM, HTN, CAD, CHF, RD/D, COPD – Disease characteristics: Rutherford, prior amputation – Lesion characteristics: LL, CTO, RVD, HxIL, TASC – Medications: ATT, statins
CARROT N/A 2019	Retrospective Cohort Japan	*PTX-eluting stent:* Zilver PTX (Cook Medical) *Control treatment:* POBA, BMS	Total: 1,535 Exposed: 285 Controls:1,250	Follow-up: 60 months 3 years: PTX: 235 (82%) Controls: 967 (77%) 5 years: PTX: 113 (40%) Controls: 383 (31%)	Safety (mortality) Limited generalizability	– Mortality outcome: all-cause, CV-related – Lifestyle: BMI, SMK – Comorbidities: DM, HTN, RD/D, COPD – Disease characteristics: Rutherford – Lesion characteristics: LL, CTO, RVD, TASC – Medications: aspirin, P2Y12 receptor blockers
PREVEIL NCT02648620 2020	Single-arm trial US	*PTX-coated balloon:* *SurVeil (SurModics)* ^c^	Exposed: 13	Follow-up: 36 months	Feasibility (peak paclitaxel plasma concentration)	– Mortality outcome: all-cause – Lifestyle: SMK – Comorbidities: DM, HTN, CHF – Disease characteristics: Rutherford
IN.PACT Global Clinical Study NCT01609296 2020	Single-arm trial 27 countries	*PTX-coated balloon:* IN.PACT Admiral (Medtronic)	Exposed:1,406	Follow-up: 60 months 3 years: 1,252 (89%) 5 years: Not available	Efficacy (TLR) Limited generalizability	– Mortality outcome: all-cause, CV-related – Lifestyle: BMI, SMK – Comorbidities: DM, IDDM, HTN, CHD, RD/D – Disease characteristics: Rutherford, prior amputation – Lesion characteristics: LL, CTO, RVD, HxIL, TASC – Medications: ATT, aspirin, P2Y12 receptor blockers, statins
Zilver PTX US PAS NCT01901289 2021	Single-arm trial US	*PTX-eluting stent:* Zilver PTX (Cook Medical) * *	Exposed: 200	Follow-up: 60 months 3 years: unknown	Effectiveness (TLR) Generalizable	– Mortality outcome: All -cause – Lifestyle: SMK – Comorbidities: DM, IDDM, HTN, CAD, CHF, RD/D, COPD – Disease characteristics: Rutherford, prior amputation – Lesion characteristics: LL, CTO, RVD, HxIL, TASC – Medications: ATT, aspirin, P2Y12 receptor blockers
ILLUMENATE GLOBAL and ISR NCT01927068 2022	Single-arm trial 10 countries	*PTX-coated balloon:* Stellarex (Spectranetics) * *	Exposed: 500	Follow-up: 60 months 3 years: Not available	Safety and Efficacy (primary patency, safety composite^f^) Limited generalizability	– Mortality outcome: All-cause – Lifestyle: SMK – Comorbidities: DM, HTN, CAD, CHF, RD/D, COPD – Disease characteristics: Rutherford – Lesion characteristics: LL, CTO, HxIL
The Efficacy of Endovascular Treatment in FPOD With TASC C and D Lesions NCT04698304 2025	Prospective Cohort China	*Drug-coated balloons* *Drug-eluting stents* *Control treatment:* POBA, BMS	Total: 1,000 Exposed: unknown, recruiting	Follow-up: 36 months	Safety and Effectiveness (primary patency, MAEs, CD-TLR) Limited generalizability	– Mortality outcome: All-cause – Disease characteristics: Rutherford
FLOWER NCT04393389 2027	Single-arm Trial Germany	*PTX-coated balloon:* AcoArt Orchid, Tulip, or Litos (Acotec Scientific)	Exposed: 3,000	Follow-up: 60 months	Safety and Effectiveness (CD-TLR, safety composites^l,m^) Limited generalizability	– Mortality outcome: device- and procedure-related – Disease characteristics: Rutherford
Registries						
LEVANT 2 Continued Access Registry NCT01628159 2018	Single-arm Registry US Austria, Belgium, Germany, Switzerland	*PTX-coated balloon:* Lutonix (CR Bard) * *	Exposed: 657	Follow-up: 60 months 3 years: 592 (90%) 5 years: 573 (87%)	Safety (unanticipated device- or drug-related AEs)	– Mortality outcome: all-cause – Lifestyle: BMI, SMK – Comorbidities: DM, IDDM, HTN, CAD, CHF, RD/D – Disease characteristics: Rutherford – Lesion characteristics: LL, CTO, RVD, HxIL, TASC – Medications: ATT, aspirin, P2Y12 receptor blockers, statins
SAFE-DCB Registry NCT02424383 2019	Single-arm all-comers cohort US	*PTX-coated balloon:* Lutonix (CR Bard) * *	Exposed: 1,005	Follow-up: 36 months 3 years: 799 (80%)	Safety and Effectiveness (TLR, safety composite^f^) Generalizable	– Mortality outcome: all-cause – Lifestyle: SMK – Comorbidities: DM, HTN, CAD, CHF, RD/D – Disease characteristics: Rutherford, prior amputation – Lesion characteristics: LL, CTO, RVD, HxIL
SAVER Registry NCT02769273 2021	Single-arm all-comers cohort Austria, Belgium, France, Germany, Italy, United Kingdom	*PTX-coated balloon:* Stellarex (Spectranetics) * *	Exposed: 10,000	Follow-up: 36 months	Safety and Effectiveness (TLR, safety composite^f^)	– Mortality outcome: all-cause – Lifestyle: SMK – Comorbidities: DM, HTN, CAD, CHF, RD/D, COPD – Disease characteristics: Rutherford – Lesion characteristics: LL, CTO, HxIL
IN.PACT ISR PMS N/A 2023	Single-arm Cohort (VQI Registry) US	*PTX-coated balloon:* IN.PACT Admiral (Medtronic) * *	Exposed: 300	Follow-up: 36 months	Effectiveness (TLR) Generalizable	– Mortality outcome: all-cause – Lifestyle: BMI, SMK – Comorbidities: DM, IDDM, HTN, CAD, CHF, RD/D, COPD – Disease characteristics: Rutherford, prior amputation – Lesion characteristics: LL, CTO, RVD, HxIL – Medications: ATT, aspirin, P2Y12 receptor blockers, statins
LEGDEB2 Registry NCT04175197 2024	Single-arm all-comers cohort Italy, Mexico	*PTX-coated balloon:* Legflow (Cardionovum GmbH)^c^	Exposed: 512	Follow-up: 36 months	Safety and Effectiveness (TLR, safety composite^n^) Limited generalizability	– Mortality outcome: all-cause, CV-related – Lifestyle: SMK – Comorbidities: DM, HTN – Disease characteristics: Rutherford – Lesion characteristics: LL, CTO, HxIL – Medications: aspirin, P2Y12 receptor blockers
ELEGANCE Registry NCT04674969 2028	Cohort all-comers US	*PTX-coated balloons:* RANGER (Boston Scientific) *PTX-eluting stents:* Eluvia (Boston Scientific)	Exposed: 5,000	Follow-up: 60 months	Safety and Effectiveness (primary patency, safety composite^o^) Generalizable	– Mortality outcome: all-cause
LUMIFOLLOW Registry NCT04743180 2026	Cohort all-comers France	*PTX-coated balloons:* LUMINOR * *	Exposed: 500	Follow-up: 60 months	Safety and Effectiveness (primary patency, safety composite^p^) Limited generalizability	– Mortality outcome: all-cause, CV-related – Disease characteristics: Rutherford – Lesion characteristics: LL, CTO, TASC
VISION Coordinated Registry Network (VQI PVI Registry) N/A Ongoing study	VQI PVI Prospective registry linked to Medicare claims, German Administrative Claims, State-based Claims US	*PTX-coated balloons:* IN.PACT Admiral (Medtronic) Lutonix (CR Bard) Stellarex (Spectranetics) *PTX-eluting stents:* Zilver PTX (Cook Medical) Eluvia (Boston Scientific) *Control treatment:* POBA, BMS	Total: ∼15,000 Exposed: ∼6,000	Follow-up: 60 months	Safety (mortality) Generalizable	– Mortality outcome: all-cause – Lifestyle: BMI, SMK – Comorbidities: DM, IDDM, HTN, CAD, CHF, RD/D, COPD – Disease characteristics: Rutherford, prior amputation – Lesion characteristics: LL, CTO, RVD, HxIL – Medications: ATT, aspirin, P2Y12 receptor blockers, statins
SAFE PAD CMS NCT04496544 2023	Medicare claims US	*PTX-coated balloons:* IN.PACT Admiral (Medtronic) Lutonix (CR Bard) Stellarex (Spectranetics) *PTX-eluting stent:* Zilver PTX (Cook Medical) *Control treatment:* POBA, BMS	Total: 250,000	60 months 3.4 years: 25,000 (17%)	All-cause mortality signal Medicare-insured population	– Mortality outcome: all-cause – Lifestyle: BMI, SMK – Comorbidities: DM, HTN, CHF, RD/D, COPD – Disease characteristics: Rutherford
State of New York Claims Ongoing study	Administrative claims US	*Any PTX device* *Control treatment:* POBA, BMS	Total: ∼4,000 Exposed: ∼1,600 Controls: ∼2,400	Follow-up: 36 months	Effectiveness (amputation, reintervention) Generalizable	– Mortality outcome: in-hospital all-cause – Comorbidities: DM, HTN, CAD, CHF, RD/D, COPD – Disease characteristics: prior amputation – Lesion characteristics: HxIL
State of California Claims Ongoing study	Administrative claims US	*Any PTX device* *Control treatment:* POBA, BMS	Total: ∼5,500 Exposed: ∼2,200 Controls: ∼3,300	Follow-up: 36 months	Effectiveness (amputation, reintervention) Generalizable	– Mortality outcome: in-hospital all-cause – Comorbidities: DM, HTN, CAD, CHF, RD/D, COPD – Disease characteristics: prior amputation – Lesion characteristics: HxIL
BARMER—Freisinger N/A 2017	Administrative claims Germany	*PTX-coated balloons* *PTX-eluting stents* *Control treatment:* POBA, BMS	Total: 64,771 Exposed: 3,324 Controls: 61,447	Follow-up: 132 months	Safety (mortality) Limited generalizability	– Mortality outcome: all-cause – Lifestyle: BMI, SMK – Comorbidities: DM, IDDM, HTN, CAD, CHF, RD/D – Disease characteristics: Rutherford, prior amputation – Lesion characteristics: HxIL
BARMER—Behrendt NCT03909022 2026	Administrative claims Germany	*PTX-coated balloons* *PTX-eluting stents* *Control treatment:* POBA, BMS	Total: 37,914 Exposed: 10,773 Controls: 27,141	Follow-up: 96 months 3 years: PTX: 3,463 (32%) Controls: 3,379 (12%) 5 years: PTX: 1,142 (11%) Controls: 1,206 (4%)	Prevalence of the outpatient prescription of best medical treatment ^q^ Limited generalizability	– Mortality outcome: all-cause – Lifestyle: BMI, SMK – Comorbidities: DM, IDDM, HTN, CAD, CHF, RD/D, COPD – Medications: ATT, antiplatelets, oral anticoagulation
Administrative Claims						
Optum Claims N/A 2019	Administrative claims US	*PTX-coated balloons:* IN.PACT Admiral (Medtronic) Lutonix (CR Bard) Stellarex (Spectranetics) *PTX-eluting stents:* Zilver PTX (Cook Medical) Eluvia (Boston Scientific)	Exposed: 20,536	Follow-up: Up to 45 months	Safety (all-cause mortality) Lower-risk, younger population who are primarily enrolled in private insurance or Medicare Advantage	– Mortality outcome: all-cause – Lifestyle: SMK – Comorbidities: DM, HTN, CAD – Disease characteristics: prior amputation
Real-World Safety Analysis of PTX Devices Used for the Treatment of PAD NCT04647643 2021	FAIR Health data warehouse claims US	*PTX-coated balloons* *PTX-eluting stents* * *	Exposed: 20,000	Follow-up: 48 months	Safety (all-cause mortality) Generalizable	– Comorbidities: CAD, RD/D
Electronic Health Records						
PCORNet /MDEpiNet pilot N/A Ongoing	US	*Any PTX device* *Control treatment:* POBA, BMS	Total: ∼600	Follow-up: Up to 4 years	Effectiveness (amputation, reintervention)	– Lifestyle: BMI – Comorbidities: DM, IDDM, HTN, CAD, CHF, RD/D, COPD – Disease characteristics: prior amputation – Medications: ATT, aspirin, P2Y12 receptor blockers, statins

AE, adverse events; ATT, antithrombotic therapy; BMI, body mass index; BMS, bare metal stent; CV, cardiovascular; DCB, drug-coated balloon; DES, drug-eluting stent; MAE, major adverse events; MAUDE, manufacturer and user facility device experience; OUS, outside the United States; PFPC, polymer-free paclitaxel coating; POBA, plain old balloon angioplasty; PTA, percutaneous transluminal angioplasty; PTX, paclitaxel; RCT, randomized clinical trial; TLR, target lesion revascularization; TVR, target vessel revascularization; VQI, vascular quality initiative.

^a^ ClinicalTrials.gov Identifier.

^b^ Data elements of interest are: Lifestyle (body mass index (BMI)/obesity, smoking status (SMK)); Comorbidities (diabetes mellitus (DM), insulin-dependent DM (IDDM), hypertension (HTN), coronary artery disease [e.g., history of MI, angina, etc. (CAD)], coronary heart disease (CHD), history of heart failure (CHF), renal disease/on dialysis (RD/D), chronic obstructive pulmonary disease (COPD)); Disease characteristics (Rutherford classification, prior amputation); Lesion characteristics (Lesion length (LL), chronic total occlusion (CTO), prior intervention of lesion (HxIL), reference vessel diameter (RVD), Trans-Atlantic Inter-Society Consensus (TASC) classification; and Medications at discharge.

^c^ Not approved for commercial use in the United States as of November 2021.

^d^ MAEs of death, TLR, target limb ischemia requiring surgical intervention (bypass or amputation of toe, foot or leg), surgical repair of the target vessel.

^e^ Freedom from death through 30 days or target limb major amputation or clinically-driven (CD) TVR within 12 months post index procedure (e.g., dissection requiring surgery), and from worsening of the Rutherford classification by 2 classes or to class 5 or 6.

^f^ Freedom from device- and procedure-related death through 30 days post-procedure, and freedom from target limb major amputation and CD-TVR.

^g^ Freedom from all-cause peri-operative (≤30 day) death and freedom at 1 year from the following: index limb amputation (above or below the ankle), index limb re-intervention, and index-limb-related death.

^h^ Freedom from target limb major amputation and CD-TVR through 24 months post-procedure.

^i^ Freedom from all-cause death through 1 month, target limb major amputation (defined as at or above the ankle) within 12 months, and/or TLR within 12 months.

^j^ Freedom from device- and procedure-related death through 12 months post procedure as well as freedom from both target limb major amputation and CD-TVR.

^k^ Freedom from all-cause peri-procedural (≤30 day) death and freedom at 1 year from the following: index limb amputation (above or below the ankle) and index limb re-intervention.

^l^ Freedom from major adverse limb events and perioperative death (MALE-POD) through 30 days after index procedure.

^m^ Freedom from device- and procedure-related mortality, freedom from major target limb amputation and TLR within 12 months post-index procedure.

^n^ Freedom from device- and procedure-related mortality through 30 days, from device or procedure-related mortality, from any cardiac or CV death, and from major target limb amputation.

^o^ MAEs, which include Target Lesion Revascularizations, Major Target Limb Amputations, and Deaths.

^p^ Freedom from all-cause peri-procedural (≤30 day) death and freedom at 3 years from the following: index limb amputation (above or below the ankle), and all-cause mortality (with a detailed analysis of CV and non-CV deaths).

^q^ Defined as picking up a medication at a pharmacy for a lipid-lowering, an antithrombotic, and an antihypertensive drug agent, within 12 months after index discharge for peripheral arterial occlusive disease according to information provided in health insurance claims data.

### 3.1 Identified studies

#### 3.1.1 RCTs leading to device approval

Eight brands of paclitaxel-coated devices were evaluated or are currently being evaluated in 14 premarket RCTs (Table 1), of which 7 RCTs were conducted outside of the US (OUS). The total sample size of these trials ranged between 100 and 532 subjects. The number of patients treated with paclitaxel-coated devices in these RCTs ranged from 48 to 524 subjects. Four trials compared DCBs vs. DCBs, DCBs vs. DES, and DES vs. DES. The remaining randomized subjects received paclitaxel-coated devices or plain old balloon angioplasty (POBA). The majority of these RCTs (*n* = 11) had primary endpoints for the safety and efficacy of these devices, and the same number have a follow-up duration of five years.

#### 3.1.2 RCTs conducted postmarket

Four European postmarket RCTs, including two registry-based RCTs, were identified (Table 1). The studies evaluated two FDA-approved DES and paclitaxel-coated devices in Sweden. These postmarket RCTs included more patients (220–2,400 subjects) than the premarket studies with up to 1,200 subjects exposed to a paclitaxel-coated device. The majority of the trials evaluate effectiveness as the primary endpoint. All trials have a follow-up duration of 5 years and are expected to be completed in one to five years or by 2025 at the latest.

#### 3.1.3 Single-arm and cohort studies

Paclitaxel-coated devices were evaluated in two single-arm US studies and four OUS studies (Table 1). Half of these studies include safety and effectiveness as primary endpoints or have a follow-up duration of 5 years. The sample sizes range between 13 and nearly 1,500 subjects. One study evaluated a DCB not approved for commercial use in the US (as of August 2020). A retrospective cohort study examined all-cause mortality comparing DES (*n* = 285) with non- paclitaxel-coated devices (POBA or bare-metal stents, *n* = 1,250) in Japan was also identified. This cohort study had a median follow-up of 3.4 years (interquartile range: 2.1, 5.7).

### 3.2 Identified data sources

A total of 7 distinct registry-based RCTs were identified. The data sources included information on seven brands of FDA-approved DCBs (three approved for commercial use in the US) and two brands of FDA-approved DESs. In addition to the registry-based RCTs identified, one coordinated registry network linking five data sources capturing vascular procedures internationally was identified. Three additional data sources captured private or commercial state and national level administrative claims as well as electronic health records.

### 3.3 Quality assessment

Almost all data sources capture either all-cause or cardiovascular (CV)-related mortality in regular intervals of 1, 6, 12, 24, and 36-month intervals. Most data sources only present aggregate data and rarely make patient-level data available. Given that most data sources are clinical trials, patient records beyond what is collected are often not accessible. Quality assessments of the included studies and data sources are summarized in Table 2.

**Table 2:** Quality Assessment of data sources for paclitaxel signal Discernment.

Study/ Data Source Identifier Completion Date	Critical data element availability			Study Design	Data Collection: Quality Assessment			
	Outcome (mortality: all cause and CV-related)	Lifestyle variables—available in data (BMI, smoking, alcohol use)	Comorbidities—available in data?		Any notable changes in variable capture by site or over time?	Average time between points of data capture	Access to data—patient level data available, aggregated level data only, other?	Ability to go back to clinical records?
Thunder NCT00156624 2007	All-cause	Yes	Yes	Randomized Clinical Trials (Premarket)	Follow-up was not included in the original study protocol.	12, 24, and 60 months	Aggregated level data	Unclear
Zilver PTX NCT00120406 2014	All-cause	Yes	Yes	Randomized Clinical Trials (Premarket)	None Found	6 months, 12 months, and at 2, 3, 4, and 5 years	Aggregated level data	Unclear
REAL PTX NCT01728441 2017	All-cause and CV-related	Yes	Yes	Randomized Clinical Trials (Premarket)	Changes in primary outcomes and follow-up was changed from 12 months to at least 36 months.	6, 12, and 24 months	Aggregated level data	Yes
IN.PACT SFA I and SFA II NCT01175850 NCT01566461 2018	All-cause	Yes	Yes	Randomized Clinical Trials (Premarket)	None Found	1 and 12 months	Aggregated level data	Unclear
IN.PACT SFA Japan NCT01947478 2018	All-cause	Yes	Yes	Randomized Clinical Trials (Premarket)	None Found	1, 6 and 12 months	Aggregated Data	Yes
LEVANT 2 NCT01412541 2018	All-cause	Yes	Yes	Randomized Clinical Trials (Premarket)	None Found	6, 12, and 24 months	Aggregated Data	Unclear
RANGER SFA NCT02013193 2019	All-cause within 6 months	Yes	Yes	Randomized Clinical Trials (Premarket)	None Found	6 months	Patient-level Data may be available	Yes
ILLUMENATE EU RCT NCT01858363 2020	All-cause and CV-related	Yes	Yes	Randomized Clinical Trials (Premarket)	None Found	30 days, 1, 6, 12, 24 months	Patient-level Data may be available	Yes
ILLUMENATE RCT/PAS NCT03421561 2020	All-cause and CV-related	Yes	Yes	Randomized Clinical Trials (Premarket)	None Found	24,36,48 and 60 months	Patient-level Data may be available	Yes
IMPERIAL NCT02574481 2022	All-cause	Yes	Yes	Randomized Clinical Trials (Premarket)	None Found	12 months	Aggregated Data	Unclear
RANGER II SFA NCT03064126 2023	All-cause	Yes	Yes	Randomized Clinical Trials (Premarket)	None Found	6 and 12 months	Patient-level Data may be available	Unclear
Compare I NCT02701543 2023	All-cause	Yes	Yes	Randomized Clinical Trials (Premarket)	None Found	6, 12, 24, 36, 48 and 60 months	Patient-level Data may be available	Unclear
TRANSCEND NCT03241459 2024	All-cause	Yes	Yes	Randomized Clinical Trials (Premarket)	None Found	1, 6, 12, 24,36,48 and 60 months	No results available	Unclear
XPEDITE NCT02936622 2024	All-cause	Unknown	Unknown	Randomized Clinical Trials (Premarket)	None Found	6 months	No results available	Unclear
SIRONA NCT04475783 2028	All-cause	Unknown	Unknown	Randomized Clinical Trials (Premarket)	None Found	1, 6, 12, 24, 36, 48 and 60 months	No results available	Unclear
SWEDEPAD 1 NCT02051088 2021	All-cause	Yes	Yes	Randomized Clinical Trials (Postmarket)	Added Follow-up time at 3 and 5 years	30 days, 12, 36, 60 months	Patient-level Data may be available	Yes
SWEDEPAD 2 NCT02051088 2021	All-cause	Yes	Yes	Randomized Clinical Trials (Postmarket)	Added Follow-up time at 3 and 5 years	30 days, 12, 36, 60 months	Patient-level Data may be available	Yes
ZilverPass NCT01952457 2022	All-cause	Yes	Yes	Randomized Clinical Trials (Postmarket)	None Found	30 days, 12, 24, 36 months	Patient-level Data may be available	Unclear
EMINENT RCT NCT02921230 2025	All-cause	Yes	Yes	Randomized Clinical Trials (Postmarket)	None Found	1, 6, 12, 24, 36, 48, and 60 months	Patient-level Data may be available	Unclear
Zilver PTX and Flex Japan PMS NCT02254837 2018	All-cause	No	Yes	Single-Arm and Cohort Studies	None Found	12, 24, and 60 months	Patient-level Data may be available	Yes
Lutonix DCB Long Lesions NCT02013271 2018	All-cause	Yes	Yes	Single-Arm and Cohort Studies	Added Secondary Endpoints and Changed to Observational from Interventional	1, 6, 12, 24, 36 months	No results available	Unclear
CARROT N/A 2019	All-cause and CV-related	Yes	Yes	Single-Arm and Cohort Studies	None Found	12, 36, 60 months	Aggregate	No
PREVEIL NCT02648620 2020	All-cause	Yes	Yes	Single-Arm and Cohort Studies	None Found	1, 6, 12, 24, 36 months	No results available	Unclear
IN.PACT Global Clinical Study NCT01609296 2020	All-cause and CV-related	Yes	Yes	Single-Arm and Cohort Studies	None Found	1, 6, 12, 24, 36, 48, and 60 months	Patient-level Data	Unclear
Zilver PTX US PAS NCT01901289 2021	All-cause	Yes	Yes	Single-Arm and Cohort Studies	None Found	12, 24, 36, 48, and 60 months	Patient-level Data	Unclear
ILLUMENATE GLOBAL and ISR NCT01927068 2022	All-cause	Yes	Yes	Single-Arm and Cohort Studies	None Found	1, 12, 36, 48, 60 months	Patient-level Data	Unclear
The Efficacy of Endovascular Treatment in FPOD With TASC C and D Lesions NCT04698304 2025	Not Stated	Not Stated	Not Stated	Single-Arm and Cohort Studies	None Found	7 days, 24 months, 36 months	No results available	Unclear
FLOWER NCT04393389 2027	Device—Procedure-related Mortality	Not Stated	Not Stated	Single-Arm and Cohort Studies	None Found	1, 6, 12, 24, 36, 48, 60 months	No results available	Unclear
LEVANT 2 Continued Access Registry NCT01628159 2018	All-cause	Yes	Yes	Registries	Added Secondary Outcomes	1, 6, 12, 24, 36, 48, and 60 months	Patient-level Data	Unclear
SAFE-DCB Registry NCT02424383 2019	All-cause	Yes	Yes	Registries	None Found	1, 6, 12, 24, and 36 months	Aggregate Data	Unclear
SAVER Registry NCT02769273 2021	All-cause and CV-related	Yes	Yes	Registries	None Found	1, 12, 36 months	Aggregate Data	No
IN.PACT ISR PMS N/A 2023	All-cause	Yes	Yes	Registries	None Found	12, 24, and 36 months	Aggregate Data	Unclear
LEGDEB2 Registry NCT04175197 2024	All-cause and CV-related	Yes	Yes	Registries	None Found	1, 12, 24, 36 months	Patient-level Data	No
ELEGANCE Registry NCT04674969 2028	All-cause	Not Stated	Not Stated	Registries	None Found	12 months	Unclear	Unclear
LUMIFOLLOW Registry NCT04743180 2026	All-cause and CV-related	Not Stated	Not Stated	Registries	None Found	1, 6, 12, 36, 48, 60 months	No results available	Unclear
VISION Coordinated Registry Network (VQI PVI Registry) N/A Ongoing study	All-Cause	Yes	Yes	Registries/Claims	None Found	Captured as billed or entered	Patient-level data not publicly available	Yes
SAFE PAD CMS NCT04496544 2023	All-Cause	Yes	Yes	Registries/Claims	None Found	Captured as billed	Patient-level data not publicly available	No
State of New York Claims Ongoing study	No	No	Yes	Registries/Claims	None Found	Captured as billed or entered	Patient level	No
State of California Claims Ongoing study	No	No	Yes	Registries/Claims	None Found	Captured as billed or entered	Patient level	No
BARMER—Freisinger N/A 2017	All-Cause	No	Yes	Registries/Claims	None Found	Captured as billed	Patient-Level Data	No
BARMER—Behrendt NCT03909022 2026	All-Cause	Yes	Yes	Registries/Claims	None Found	Captured as billed	Patient-level data not publicly available	No
Optum Claims N/A 2019	All-Cause	No	Yes	Claims	None Found	Captured as billed	Patient-Level Data	No
Real-World Safety Analysis of PTX Devices Used for the Treatment of PAD NCT04647643 2021	No	No	Yes	Claims	None Found	Captured as billed	Patient-Level Data	No
PCORNet /MDEpiNet pilot N/A Ongoing	No	Yes	Yes	Electronic Health Records	None Found	Captured as billed or entered	Patient-Level Data	No

### 3.4 Identified data-driven methodologies

Given the extracted studies, data sources, and their respective limitations, methodologies focusing on the use of existing premarket clinical data, the use of real-world data (RWD) to overcome RCT limitations (i.e., lost to follow-up), approaches for individual-level data, machine learning and artificial intelligence approaches, Bayesian approaches, and the combination of various datasets were summarized.

## 4 Discussion

The review identified 39 studies and data sources that can aid in the signal detection of paclitaxel DCBs and DESs. While RCTs provide critical information to regulatory bodies prior to approval and, have the potential to produce high-quality, detailed data and have minimal risk of introducing confounding due to randomization, they suffer from critical limitations. Trials, however, are limited to specific study populations, may greatly differ in eligibility criteria for included patients between studies, have low external validity, have short follow-up periods and are plagued by possible high rates of loss to follow-up. If a device is authorized, its application and performance in clinical practice must be continuously assessed, under both the existing and broader conditions of use, to detect any potential safety and effectiveness signals promptly. High-quality RWD, as characterized by the FDA (5), employing appropriate statistical methods, summarized below, can build upon data collected from premarket studies and provide a more comprehensive and continuous assessment of devices.

### 4.1 Existing real-world data sources for the assessment of paclitaxel-coated devices

#### 4.1.1 Strategically coordinated registry networks (CRNs)

Coordinated Registry Networks (CRNs) create a robust and comprehensive source for medical device evaluation by growing existing data sources' capacity through the organization and linkage data systems to circumvent the limitations of individual data sources and create a robust and comprehensive source for medical device evaluation (10, 11). As with all databases, the quality and capability of a registry play a vital role in its ability for accurate and timely evaluations. Robust registries continuously and consistently collect data relevant to multiple stakeholders, including patients, physicians, manufacturers, and regulatory bodies. It is paramount that registries be generalizable to the population utilizing the medical device and afford evaluation of meaningful outcomes that improve the quality of patient care (12). Registries that incorporate standardized data elements and standardized libraries for device identification, such as the Fast Healthcare Interoperability Resources (FHIR) and unique device identifiers (UDI), should be employed. The standardization of data elements and device identifiers improves interoperability with other data sources and device identification capabilities. High-quality registries have numerous advantages. They can capture a large number and variety of procedures and devices, reflect current medical practice, have high external validity, and have the potential for long follow-up times to assess devices over their total product life cycle. However, some limitations include that individuals in registries are not randomized. In addition, limited demographic and clinical data may be collected on individuals in the registry. The risk of confounding in analyses may, therefore, be increased. High-quality registries can be linked to several claims databases, such as the Center for Medicare and Medicaid Services (CMS) claims and the Statewide Planning and Research Cooperative System (SPARCS). Claims complement registries by collecting comprehensive patient-level characteristics, diagnoses, treatments, hospitalizations, and charges for inpatient as well as outpatient services. Thus allowing researchers to evaluate all reported events or diagnoses that are related and unrelated to the medical device.

The Vascular Implant Surveillance & Interventional Outcomes Network (VISION) CRN captures detailed demographic and clinical data of patients who undergo vascular procedures with the ultimate goal of improving the quality, safety, effectiveness, and cost of vascular healthcare. VISION covers 605,322 patients in the VQI registry from over 600 academic and community hospitals across the US and Canada (13). To augment the VISION-CRN, the Vascular Quality Initiative (VQI) registry captures mortality through follow-up data submitted by providers and linkage to the social security index data. It is continuously linked to Medicare data provided by the Centers for Medicare & Medicaid Services (CMS) claims, a nationally representative dataset of Medicare-insured individuals above the age of 65 covered by FFS Medicare (14). The registry is also continuously linked to state and city representative datasets, including the California and New York Statewide Planning and Research Cooperative System (SPARCS) dataset and the New York City Clinical Data Research Network (NYC-CDRN) dataset (15).

International efforts of the VISION-CRN include the International Consortia of Vascular Registries (ICVR), which has direct data sharing from national registries in 13 countries and distributed systems for research and surveillance. ICVR continues to engage in international collaborations to perform studies within health insurance claims and registry data, such as the German administrative claims database. These analyses include thousands of health insurance claims, survival data, and event outcomes occurring between 2007 and 2017.

### 4.2 Administrative claims databases

Health insurance claims can be leveraged to identify and study paclitaxel-coated devices among commercially insured patients. Claims produce procedure codes in the form of current procedural terminology (CPT) and International Classification of Diseases (ICD) codes that only identify whether a medical device-related procedure was performed. It is important to recognize that these codes are input for billing purposes and not research purposes. Unlike national drug codes (NDC) that can identify medications by type, formulation, and dose, CPT codes are not granular and cannot identify which specific medical device was used. Despite the lack of granularity in the identification of the device, claims have the potential to follow individuals over a long period of time, allowing for the evaluation of long-term outcomes throughout the product's life cycle. Optum and FAIR Health Data are two administrative claims data repositories of over 20,000 patients within the US that follow patients for up to 48 months. The available data either captures all-cause mortality directly or relies on linkages with vital statistics to capture mortality. It is important to note that all-cause death and date of death may be missing for patients who are no longer captured by the dataset because they changed insurance plans or are no longer eligible for a specific type of insurance.

### 4.3 Electronic health records—based studies

#### 4.3.1 PCORNet /MDEpiNet pilot

The National Patient-Centered Clinical Research Network (PCORNet) captures and combines Electronic Health Record (EHR) data from multiple institutions within a given area. The captured data permit the identification of procedures and provide information on follow-up visits within a network of institutions. The New York City Insight Clinical Research Network gathers EHR data from five major hospitals in the city. Implanted paclitaxel devices can be identified using the Healthcare Common Procedure Coding System (HCPCS). Follow-up data regarding the patients receiving these interventions can be examined using PCORNet data.

#### 4.3.2 Data-driven methodologies for the assessment of paclitaxel-coated devices

High-quality data are available and accessible, though still useful in many other aspects of medical-device-related research, and may not be sufficient to properly detect the signals needed to raise regulators a device's performance. Appropriate statistical analyses tailored to the type, amount, and elements available in data sources need to be employed to take advantage of a data source's capabilities and accurately identify any potential signals (16).

### 4.4 Leveraging existing premarket clinical data

Data from RCTs may be utilized to assess devices not only in the premarket phase but also postmarket. RCTs may be utilized to identify potential signals when real-world data (RWD) capturing the devices of interest are not yet available. One may initially fill the gaps with existing data that has already been analyzed. While randomization generally provides balance at the baseline between two alternatives (e.g., devices with and without paclitaxel), missing visits or loss-to-follow-up within an RCT over time can lead to unbalanced groups in the assessment of long-term outcomes (e.g., mortality 3–5 years after initial procedure). Thus, analyzing the RCTs as RWE cohorts may elucidate important, data-driven factors affecting the outcome. For such analyses, accounting for both time-varying factors and competing risks (e.g., loss to follow-up due to death) is essential. Additionally, accounting for differences in patients who were and were not included in RCTs is crucial. The separate evaluation of patients in RWE studies who would have met RCT inclusion/exclusion criteria vs. those who were ineligible may enlighten researchers regarding critical differences between those included and excluded from RCTs, important confounders, timing of outcomes, and other interactions with healthcare.

### 4.5 Incorporating all contributed patient time

Time-to-event statistical models are beneficial for assessing long-term outcomes of medical devices in RWD because they consider the entirety of patient-contributed time. These methods mitigate the effects of loss to follow-up and allow individuals with varying follow-up times who may or may not have experienced an outcome of interest to contribute to the analysis.

### 4.6 General approaches for aggregated data

• When aggregated data are the only data available, then traditional meta-analytic approaches that combine data across studies comparing the same treatments are commonly used to generate estimates. Thus, estimates from studies comparing the control, plain old balloon angioplasty (POBA), with paclitaxel-coated devices can be combined to provide a new (combined) device effect. A network meta-analysis combines treatment estimates that have been compared within a study (called a direct estimate) to provide a more precise device effect estimate but also undertakes indirect comparisons of two different devices that have used the same comparator but have not been compared head-to-head. Cross-design synthesis involves combining effect estimates from randomized trials with strong internal validity with observational studies with strong external validity. All of these approaches are appropriate for determining estimates using distributed computing systems.

### 4.7 General approaches for individual-level data

When individual data are available, more flexibility in estimating device effects is possible, as well as a greater ability to assess the required statistical assumptions. For instance, when interest focuses on determining if patient characteristics modify device performance, individual-level data provide more power to identify the interaction than aggregated data. Assumptions about transitivity, consistency, validity, and selection bias are still required. Due to heterogeneity in the data sources, such as different clinical trials, different database registries, different countries, random effects for each data source are virtually always required.

### 4.8 Machine learning and artificial intelligence approaches

With the expansion and growing amount of RWD, researchers can utilize artificial intelligence approaches such as machine learning to better understand and, in turn, predict how patient, clinical, and device-related factors may influence decisions relating to procedures and relevant outcomes (17). Machine-learning-based models have several advantages over traditional regression-based models (18) and may thus generate robust predictive models that can predict when a risk of a particular outcome, such as late-stage mortality, is higher among patients treated with specific devices, including paclitaxel-coated devices.

### 4.9 Bayesian approaches

Bayesian techniques provide a natural way to integrate RWD and RCTs, allowing the incorporation of prior information and providing flexible yet interpretable models. Several strategies have been developed to incorporate such studies to provide robust predictive models (19). These include using the so-called power priors (20), commensurate priors (21), and the considered “gold standard” of hierarchical modeling (22, 23). More recent techniques integrate patient-level information from observational studies and previous trials as synthetic or external controls, using confounding adjustment methods (24–26). Bayesian methods have the potential to provide a more comprehensive understanding of long-term mortality for paclitaxel-coated devices.

### 4.10 Additional methods

Data integration from different multinational studies implies the need for parsing the risks posed by various genetic and environmental factors that can affect underlying conditions, treatment choices, and ultimately treatment-related health outcomes. Many populations from the pivotal studies cited in Table 1 are expected to differ in demographics, including socioeconomic and other race/ethnicity-related characteristics. Black race/ethnicity, for instance, has been associated with increased risk of peripheral arterial disease, an underlying condition for the use of DCB and DES (27). PAD has been associated with two SNPs—Single Nucleotide Polymorphisms (28) both of which demonstrate race/ethnicity-related differences in their risk allele populational frequencies (e.g., Africans vs. Europeans/Caucasians) (27, 28). In general, genetic risk assessment of device-related adverse outcomes in patient subpopulations requires laborious efforts on biomarker discovery and validation, which are beyond the scope of the currently proposed research methodology. However, when partitioning the race/ethnicity-related risk factors in this endeavor, it is important to consider a complex, and in some cases opposing, interplay of genetic and environmental components, instead of anticipating a negative summation or potentiation of socioeconomic and genetic effects in the ethnic minority patients.

### 4.11 Combining evidence types

**Figure 1:**
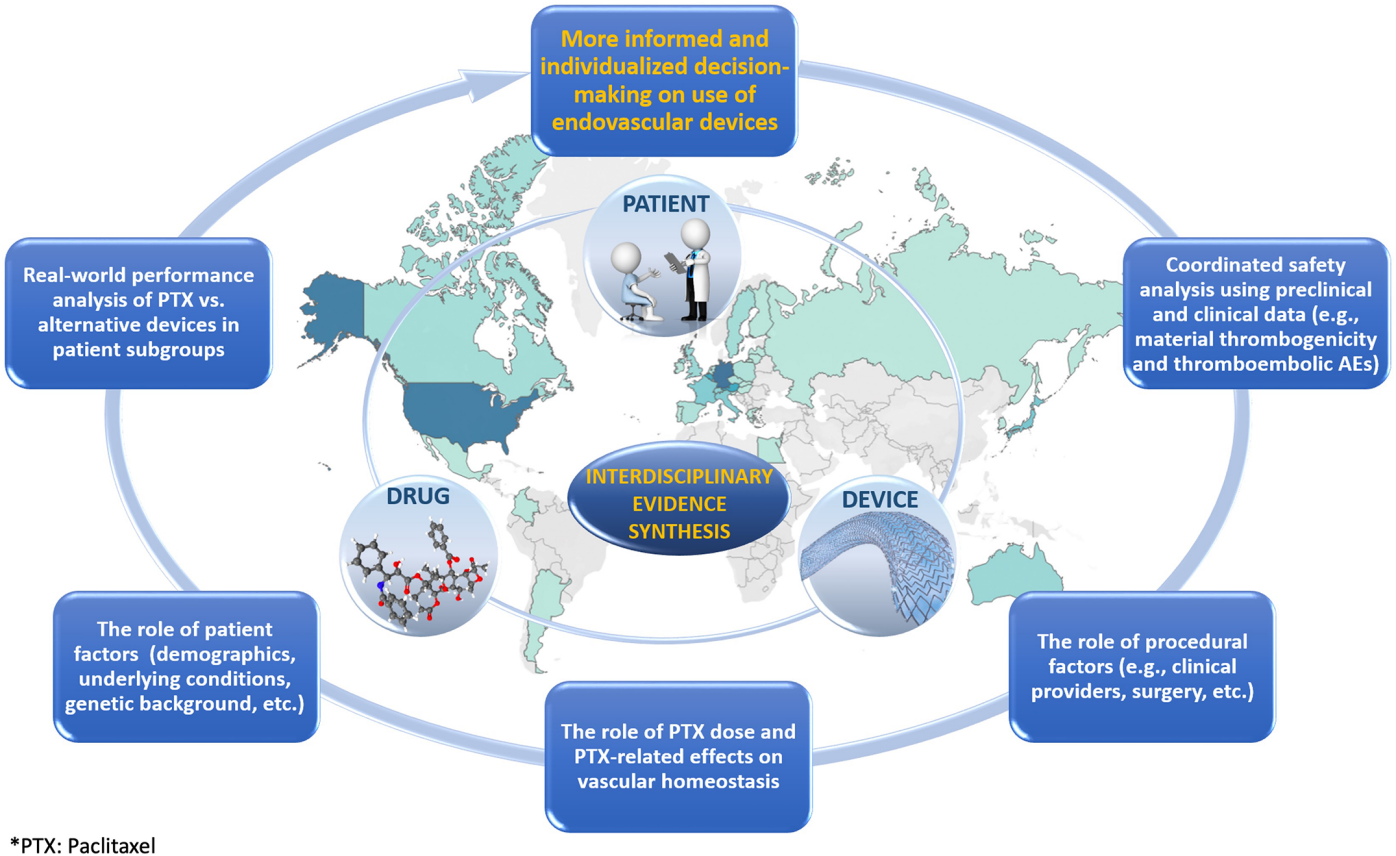
Graphic illustrating the role of interdisciplinary evidence synthesis in the evaluation of treatments.

Due to a multitude of potential device-, drug-, and patient-related factors contributing to the sum-effect, the presumed increase of all-cause mortality should be investigated using coherent linkage of multidisciplinary data that can transcend the disciplinary boundaries. The resultant interdisciplinary evidence is expected to move from bioengineering (device), pharmacological (drug), and epidemiological (patient) silos to promote the more comprehensive examination of potential synergistic effects that may remain undetected otherwise (Figure 1).

The original report on increased late mortality from paclitaxel-containing devices in femoropopliteal applications suggested a combined role of drug- and patient-related factors (i.e., paclitaxel dose and peripheral arterial disease in the lower limbs as an underlying condition, respectively) (3). Although not all subsequent studies (11, 29) confirmed the initial findings, the elusive risk increase was also attributed to patient-related factors such as the length of lesion as well as different comorbidities (30, 31). With the actual causes still unknown, non-target paclitaxel embolization was indicated as a plausible mechanism (32). This suggests the need for more inclusive preclinical and clinical data analyses aimed at exploring the drug-, device-, and patient-attributable modifications of thromboembolism. While the drug-related risk component in thromboembolism may include paclitaxel effects on vascular homeostasis (32), the device-related risk component may involve thrombogenicity as a possible manifestation of inflammatory vascular tissue remodeling due to device/material bioreactivity (33).

Thus, while the siloed approaches may obscure the intersectional risk of increased mortality, which is likely limited to certain patient/device subgroups, the root-cause analysis employing interdisciplinary evidence can apportion the mortality risk more adequately and, most importantly, can minimize a potential failure to recognize the complex interplay of various risk modifiers.

## 5 Conclusion

The meta-analysis that sparked the regulatory action occurred 17 years after the first clinical trial assessing a paclitaxel-coated device was initiated. Even with the multitudes of available studies reviewed within the committee assembled by the FDA, it was agreed upon that additional data were needed to comprehensively assess the late-mortality signal. While several RWD sources exist and may help further assess the safety signal produced among paclitaxel-coated devices and their relevant outcomes among greater patient populations, each data source has limitations and varies in quality (34). Combining the myriad of clinical studies, available RWD, and additional evidence types may allow for a more comprehensive assessment of the safety signal produced by paclitaxel-coated devices across the product's lifecycle and the role of patient-, device-, and drug-related factors. The amalgamation of the identified high-quality data sources with sophisticated statistical methods will allow for the generation of real-world evidence needed to identify and confirm the safety signal promptly and accurately. Thus providing the FDA with the needed high-quality evidence to make relevant and correct regulatory decisions regarding the safety of paclitaxel-coated devices.
